# Associations Between Specific Forms of Teasing and Body Image Dissatisfaction in Adolescents: A School‐Based Study

**DOI:** 10.1111/josh.70233

**Published:** 2026-09-09

**Authors:** André de Araújo Pinto, Guilherme José Silva Ribeiro

**Affiliations:** ^1^ Health Sciences Center, State University of Roraima Boa Vista Brazil; ^2^ Graduate Program in Nutrition Science, Department of Nutrition Federal University of Viçosa Viçosa Brazil

**Keywords:** adolescents, appearance, mental health, motor skills, weight stigma

## Abstract

**Background:**

Beyond traditional weight stigma, school‐based peer teasing critically drives adolescent body dissatisfaction, yet its distinct impact on both overweight and thinness concerns remains largely overlooked. This study examined associations between specific forms of teasing and both directions of body image (BI) dissatisfaction among adolescents.

**Methods:**

A school‐based survey was conducted with 1032 adolescents aged 14–19 years in Northern Brazil. Participants reported on four teasing modalities and body dissatisfaction, which were analyzed using survey‐weighted multinomial logistic regression models adjusted for sociodemographic and behavioral covariates.

**Results:**

Only 27.1% of adolescents reported BI satisfaction; 41.2% and 31.7% were dissatisfied due to overweight and thinness. All teasing forms were associated with higher risks of both outcomes. Mockery of physical appearance showed the strongest associations (overweight RRR = 2.08, 95% CI: 1.41–3.08; thinness RRR = 2.75, 95% CI: 1.83–4.15). No sex interactions were observed (*p* > 0.05).

**Implications for School Health Policy, Practice, and Equity:**

School‐based strategies should address diverse forms of teasing beyond weight stigma, including explicitly recognizing weight‐ and appearance‐based teasing within anti‐bullying policies and prevention programs.

**Conclusions:**

Multiple forms of teasing are associated with BI dissatisfaction among adolescents regardless of sex. Addressing body‐directed peer teasing may represent an important target for school health initiatives aimed at promoting healthier body perceptions.

## 1 Introduction

Body image (BI) dissatisfaction is highly prevalent during adolescence [1], a developmental period characterized by rapid physical, cognitive, and social changes [2]. Among adolescents, body perceptions are intrinsically linked to emotional well‐being, self‐esteem, and health‐related behaviors [3, 4]. Notably, when BI dissatisfaction occurs, it is frequently associated with disordered eating, depressive symptoms, and poorer quality of life [5]. This positions adolescence as a critical window for examining the determinants of BI dissatisfaction, particularly as body‐related concerns typically intensify during this period [2].

BI is shaped not only by individual perceptions but also by social experiences and interpersonal feedback [6]. During adolescence, peer relationships gain prominence and social evaluation becomes increasingly salient [7]. Within this context, the comments, reactions, and judgments of peers may communicate socially valued body norms and expectations [8, 9]. As a result, everyday peer interactions may shape how adolescents interpret and evaluate their own appearance and physical abilities [5, 7].

Teasing represents a frequent form of peer interaction in adolescent social environments, ranging from explicit mockery and appearance‐related comments to more subtle expressions of social evaluation [10, 11, 12]. Repeated exposure to body‐related teasing may reinforce sociocultural appearance standards and increase adolescents' sensitivity to external judgments [1, 13]. As peer evaluations become internalized, adolescents may engage in greater self‐monitoring and body scrutiny, processes that have been associated with BI dissatisfaction [3, 4, 8].

Although between 21% and more than 60% of adolescents report experiencing teasing, the phenomenon is often operationalized through aggregate measures that may obscure its inherent complexity, which leaves important gaps in current knowledge [1, 14]. Moreover, in many studies, different forms of teasing are combined into broad categories, making it difficult to understand how specific experiences relate to BI [8, 14]. This tendency is reflected in the strong emphasis on weight and appearance‐based teasing, while other forms of body‐related social devaluation, including mockery of poor motor coordination, remain understudied. These experiences are especially salient within school and physical education settings, where the visibility of physical performance and peer interactions may increase opportunities for social evaluation and teasing [9].

At the same time, BI dissatisfaction is frequently approached as a single construct, even though dissatisfaction due to overweight and dissatisfaction due to thinness represent distinct concerns and social pressures [8]. These conceptual patterns are further shaped by the predominance of evidence from high‐income countries, with relatively limited data from low‐ and middle‐income settings, particularly in Latin America [1]. Consequently, evidence from adolescents in this region remains limited despite important sociocultural influences on body image and peer interactions [1]. Collectively, these patterns underscore the need to clarify how discrete peer experiences become embedded in adolescents' body‐related self‐concepts, particularly in ways that give rise to dissatisfaction linked to overweight and thinness.

Grounded in sociocultural and peer‐influence perspectives, this study addressed three research questions. We examined whether distinct forms of teasing were associated with body image dissatisfaction, whether these associations differed according to dissatisfaction due to overweight or thinness, and whether they varied by sex. Accordingly, the objective of this study was to examine the associations between distinct forms of teasing and body image dissatisfaction due to overweight and thinness among adolescents. We hypothesized that each form of teasing (appearance‐related disparaging looks, appearance‐related mockery, mockery of poor motor coordination, and weight‐related name‐calling) would be positively associated with both dissatisfaction due to overweight and dissatisfaction due to thinness. We further hypothesized that these associations would be observed among both boys and girls.

## 2 Methods

### 2.1 Participants

This school‐based cross‐sectional observational study was conducted in public high schools in Boa Vista, the capital of Roraima state in northern Brazil. Boa Vista has approximately 413,000 inhabitants and a predominantly urban population. Data collection occurred from March to June 2025.

The target population comprised adolescents aged 14–19 years enrolled in public high schools in Boa Vista. According to the State Department of Education and Sports, 28,745 adolescents were distributed across nine educational districts. Eligible participants were adolescents aged 14–19 years enrolled in public state schools and present on the day of data collection. Exclusion criteria included students outside the target age range, pregnant adolescents, and those with physical or clinical conditions preventing independent questionnaire completion. Information regarding these conditions was provided by school administrators.

A two‐stage stratified cluster sampling design was used. In the first stage, the population was stratified by the nine educational districts, and the required sample size was proportionally allocated according to the number of enrolled students. The largest public high school within each district was selected to maximize participant recruitment while preserving geographic representation across all educational districts, including those with smaller school populations. In the second stage, classrooms within selected schools were randomly selected as primary sampling units. At least one classroom from each grade (first to third year of high school) was included, with additional classrooms selected when necessary to reach the required sample size. All students in selected classrooms were invited to participate.

Sample size calculation considered a population of 28,745 adolescents, 95% confidence level, 5% margin of error, expected prevalence of 50%, design effect of 2.0, and 10% allowance for potential losses, resulting in a minimum required sample of 834 adolescents. A total of 1250 consent forms were distributed to eligible students in the selected classrooms. Of these, 1051 adolescents agreed to participate, corresponding to a participation rate of 84.1%. After excluding 19 participants (one outside the age range, six pregnant, and 12 unable to complete the questionnaire independently), the final analytical sample comprised 1032 adolescents.

### 2.2 Procedure

Data collection was scheduled with school administrations and conducted during regular class hours. Questionnaires were administered in classrooms with students seated apart to ensure privacy and independent responses. Two trained researchers explained study objectives and provided standardized instructions.

Questionnaires required approximately 30 min to complete. Immediately after completion, researchers checked questionnaires to identify missing items due to oversight. When blank responses were identified, participants were informed of the omission and given the opportunity to complete the item if they wished. They were also free to leave any question unanswered. Data entry was performed by two researchers to ensure accuracy, resulting in no missing data for variables included in the final analyses. After questionnaire completion, body weight and height were measured using standardized procedures. Weight was measured using a Tanita digital scale with 100 g precision, and height was measured using a portable Sanny stadiometer with 0.1 cm precision.

All procedures were conducted by trained researchers under the supervision of a doctoral‐level investigator. Prior to the main study, a pilot study was conducted with 67 adolescents from a public school not included in the main study to evaluate the procedures and comprehension of the instruments. The test–retest interval was 3 days.

### 2.3 Instrumentation

BI was assessed using the silhouette scale proposed by Stunkard et al. [15], which has been validated for use in Brazilian adolescents [16]. The scale consists of nine silhouettes ranging from 1, representing extreme thinness, to 9, representing severe obesity. Participants were asked to indicate which figure best represented their current body and which figure represented their ideal body. Body dissatisfaction was operationalized as the discrepancy between the ideal and current body silhouettes (ideal minus current). A score of zero indicated satisfaction. Negative scores represented a desire for a larger body size (dissatisfaction due to thinness), while positive scores indicated a desire for a smaller body size (dissatisfaction due to overweight). In the pilot study, the scale showed good test–retest reliability with an intraclass correlation coefficient of 0.72 (*p* < 0.001).

Teasing experiences were assessed using four items addressing negative comments and mockery, originally proposed by Slater and Tiggemann [10]. The instrument was culturally adapted for Brazilian adolescents using standardized procedures, including translation, back‐translation, expert review, and pretesting with the target population [17]. The items assessed four distinct forms of teasing: disparaging looks (appearance‐related disparaging looks), appearance‐related mockery, mockery of poor motor coordination, and weight‐related name‐calling. Responses were recorded on a five‐point Likert scale ranging from 1, never, to 5, always. For analysis, items were dichotomized into exposure (any occurrence) and non‐exposure (never). This approach was adopted to distinguish adolescents who had experienced teasing from those who had not and to facilitate interpretation of the findings. Additionally, frequency categories presented sparse distributions in the highest response options, which could compromise estimate stability. Similar dichotomization strategies have been used in epidemiological studies of victimization, where even isolated experiences may be associated with psychosocial outcomes [2]. In the pilot study, intraclass correlation coefficients ranged from 0.895 to 0.960 across items (*p* < 0.001).

Physical activity was assessed using items from the short version of the International Physical Activity Questionnaire adapted for Brazilian adolescents [18]. Participants reported frequency and duration of moderate to vigorous physical activity during a typical week across domains such as leisure, transportation, domestic tasks, and work. Adolescents were classified as physically active when reporting an average of at least 60 min per day of moderate to vigorous physical activity [19]. Those reporting less were classified as insufficiently active. In the pilot study conducted prior to the main survey, these measures showed moderate to good reproducibility, with intraclass correlation coefficients ranging from 0.66 to 0.90 (*p* < 0.001).

Nutritional status was determined using body mass index (BMI) calculated from objectively measured weight and height. Classification followed international age‐ and sex‐specific cutoffs for underweight, normal weight, overweight, and obesity [20, 21]. Sociodemographic variables included age in years, sex, self‐reported skin color, and family income. Sex was self‐reported as male or female. Skin color was self‐reported according to Brazilian census categories including White, Black, Brown, Yellow, and Indigenous. Monthly family income was categorized as up to two minimum wages, three to five minimum wages, and six or more minimum wages. At the time of data collection, the Brazilian monthly minimum wage corresponded to BRL 1518.00 (approximately USD 292.00).

### 2.4 Data Analysis

Descriptive statistics and prevalence estimates (as percentages) summarized study variables. We examined associations between teasing and BI dissatisfaction using survey‐weighted multinomial logistic regressions, accounting for the two‐stage stratified cluster design. With ‘satisfaction’ as the reference, we modeled dissatisfaction (thinness/excess weight) through multi‐adjusted models (age, sex, school grade, family income, nutritional status, and physical activity level). Independent associations were estimated by entering all teasing variables simultaneously. Interactions between teasing experiences and sex were tested, and in the absence of statistically significant interactions (*p* > 0.05), pooled analyses were retained. Results are presented as relative risk ratios (RRR) with 95% confidence intervals (95% CI), and statistical significance was set at *p* < 0.05. All analyses were conducted using Stata version 17 (StataCorp, College Station, TX, USA).

## 3 Results

The study included 1032 adolescents, with a mean age of 16.1 years (SD 0.99) and a nearly equal distribution between sexes. Most participants self‐identified as having brown skin color and were enrolled across all 3 years of high school. Approximately 60% of the sample reported a family income of up to two minimum wages. Regarding nutritional status, more than half of the adolescents had normal weight, while nearly 40% were classified as overweight or obese. Most participants were insufficiently physically active (59.5%). Concerning BI, less than one‐third reported satisfaction, whereas dissatisfaction due to excess weight and thinness was reported by 41.2% and 31.7% of the sample, respectively (Table 1).

**TABLE 1:** Characteristics of the study sample (*n* = 1032).

Variable	Total
Age (years), mean (SD)	16.1 (0.99)
Sex, *n* (%)	
Male	521 (50.5)
Female	511 (49.5)
Self‐reported skin color, *n* (%)	
White	218 (21.1)
Black	159 (15.4)
Brown	555 (53.8)
Yellow	38 (3.7)
Indigenous	62 (6.0)
School grade, *n* (%)	
First	402 (39.0)
Second	307 (29.7)
Third	323 (31.3)
Family income, *n* (%)	
≤ 2 minimum wages	614 (59.5)
3–5 minimum wages	309 (29.9)
≥ 6 minimum wages	109 (10.6)
Nutritional status, *n* (%)	
Underweight	44 (4.3)
Normal weight	570 (55.2)
Overweight	301 (29.2)
Obesity	117 (11.3)
Physical activity level, *n* (%)	
Insufficiently active	614 (59.5)
Physically active	418 (40.5)
Body image, *n* (%)	
Satisfied	280 (27.1)
Dissatisfied due to overweight	425 (41.2)
Dissatisfied due to thinness	327 (31.7)

**FIGURE 1:**
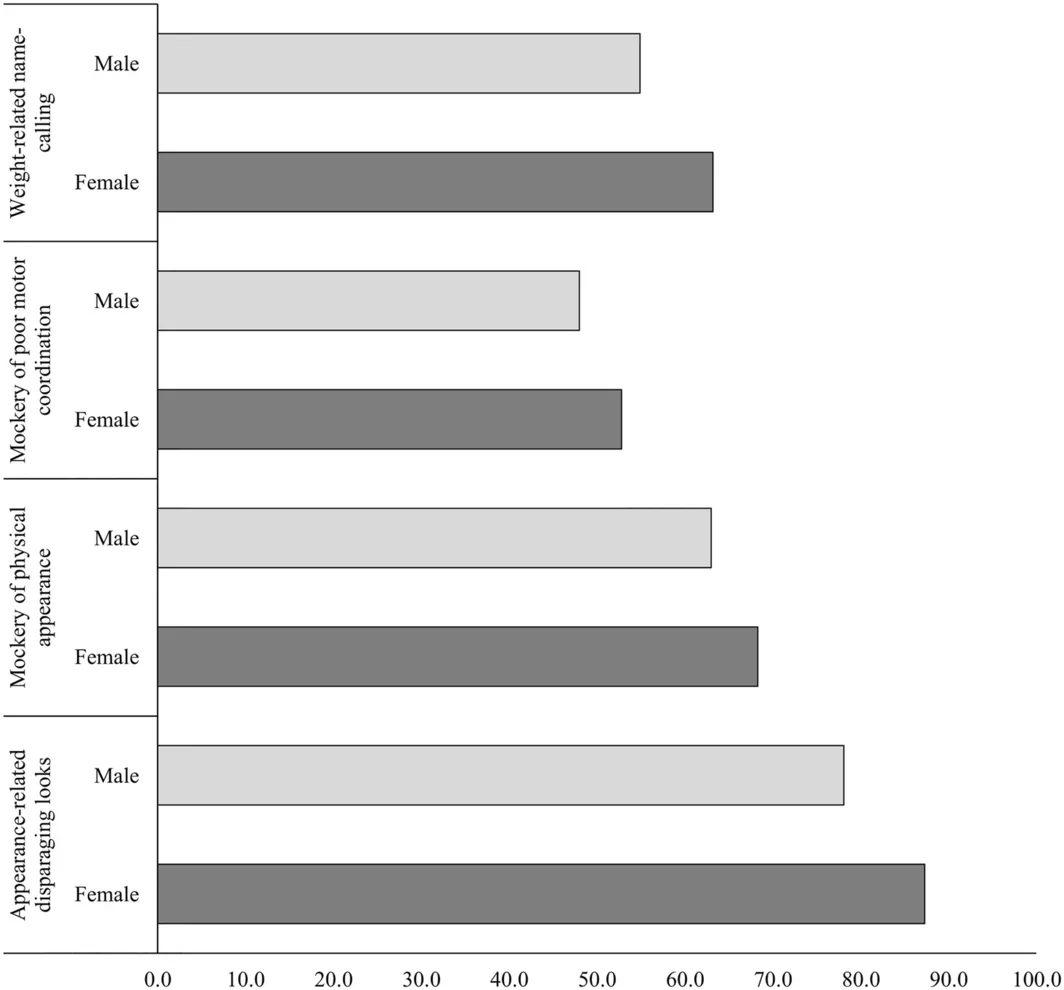
Prevalence of different types of weight‐ and appearance‐related teasing by sex.

Figure 1 shows the prevalence of four types of body‐related teasing stratified by sex. Appearance‐related disparaging looks were the most frequently reported form of teasing among both males and females, with a higher prevalence among females. Mockery of physical appearance also showed high prevalence in both sexes, again with greater reporting among females. Weight‐related name‐calling and mockery of poor motor coordination were less prevalent overall but followed a similar pattern, with females consistently reporting higher prevalence than males across all teasing forms.

Survey‐adjusted multinomial models revealed that all teasing forms were significantly associated with increased relative risk of BI dissatisfaction, whether due to thinness or overweight (Table 2). No statistically significant interactions between teasing experiences and sex were observed (*p* for interaction > 0.05), and analyses were therefore conducted with boys and girls combined. Regarding dissatisfaction due to overweight, mockery of physical appearance showed the strongest association (RRR = 2.08; 95% CI: 1.41–3.08), followed by being stared at, weight‐related name‐calling, and mockery of poor motor coordination (all *p* < 0.05). Similar patterns were observed for dissatisfaction due to thinness, where mockery of physical appearance was associated with the highest relative risk for dissatisfaction due to thinness (RRR = 2.75; 95% CI: 1.83–4.15).

**TABLE 2:** Adjusted relative risk ratios from multinomial logistic regression models examining the association between teasing experiences and body image dissatisfaction.

Teasing forms (exposure vs. non‐exposure)	Dissatisfaction due to overweight RRR (95% CI)	Dissatisfaction due to thinness RRR (95% CI)
Appearance‐related disparaging looks	1.77 (1.13–2.77)*	1.72 (1.13–2.63)*
Mockery of physical appearance	2.08 (1.41–3.08)**	2.75 (1.83–4.15)**
Mockery of poor motor coordination	1.42 (1.05–1.91)*	1.87 (1.20–2.91)*
Weight‐related name‐calling	1.57 (1.13–2.19)*	1.75 (1.07–2.85)*

*Note:* Relative Risk Ratios (RRR) and 95% confidence intervals (95% CI) were estimated using survey‐adjusted multinomial logistic regression models. Body image satisfaction was used as the reference category. All models were adjusted for age (continuous), sex, school grade, family income, nutritional status (BMI categories), and physical activity level. Teasing variables were coded as 1 = yes and 0 = no.

* *p* < 0.05.

** *p* < 0.01.

## 4 Discussion

A central finding of the present study was the consistency of associations across forms of teasing and across dissatisfaction directed toward both overweight and thinness. Although specific forms of teasing were examined separately, the convergent pattern suggests that teasing may function less as content‐specific commentary and more as an indicator of exposure to body‐directed social evaluation. This finding extends sociocultural and peer‐influence perspectives by suggesting that adolescents may internalize diverse forms of peer evaluation, rather than only appearance‐ or weight‐focused teasing, in ways that contribute to body image dissatisfaction. Notably, associations were observed not only for appearance‐ and weight‐related teasing, but also for teasing linked to perceived physical competence, underscoring the relevance of everyday interpersonal interactions in shaping adolescents' BI perceptions.

Being targeted by teasing may increase the salience of the body as an object of public scrutiny [4]. In this sense, teasing can be interpreted as a relational marker of bodily visibility within peer environments [1, 3]. Such exposure may reinforce evaluative self‐focus and body monitoring, processes that have been linked to BI dissatisfaction in adolescence [9]. Although causal pathways cannot be inferred from the present design, the consistency of associations across modalities supports the interpretation of teasing as a transversal psychosocial correlate rather than a theme‐specific influence.

Consistent with previous research, mockery of physical appearance showed the strongest associations with BI dissatisfaction [1, 4, 22]. Prior studies have repeatedly identified appearance‐ and weight‐based teasing as salient correlates of BI dissatisfaction during adolescence, reflecting the centrality of visible bodily attributes in peer evaluation [14, 23]. Although associations were observed across all forms, mockery of physical appearance showed the largest relative risk ratios, suggesting a more pronounced association between aesthetic evaluation and body dissatisfaction. This may occur because such mockery targets immediately observable characteristics [24, 25]. Consequently, appearance‐focused teasing intensifies body surveillance and self‐evaluation, as social judgment of visible traits may affect adolescents regardless of perceived thinness or heaviness [8, 14].

No significant interactions between teasing experiences and sex were observed, indicating comparable associations with BI dissatisfaction for boys and girls. This finding should be considered in the context of well‐established sex differences in BI patterns [4, 23]. Girls more frequently report dissatisfaction related to weight concerns, whereas boys often express concerns related to thinness or muscularity [6, 26]. These patterns are generally understood as reflecting distinct sociocultural body ideals [27]. Despite differences in the content of body concerns, the absence of statistical interaction suggests that, once teasing occurs, its association with dissatisfaction may operate similarly across sexes. Evidence shows that perceived physical incompetence and non‐verbal peer scrutiny, including disparaging looks, are linked to body‐related self‐perceptions, and these processes are not sex‐specific [9, 13]. The significant associations observed for teasing related to motor coordination indicate that adolescents' bodies may be evaluated not only aesthetically but also functionally within peer contexts [9], particularly in school and physical education settings [10]. Similarly, disparaging looks may function as subtle but persistent cues of social judgment, reinforcing negative self‐appraisals even in the absence of explicit verbal mockery [10, 28].

Teasing can be understood as a social experience that shapes how adolescents perceive and evaluate their own bodies [5, 11]. During adolescence, peer approval becomes highly salient [1], which is why appearance‐based comments often gain emotional weight [5]. In this context, teasing may amplify everyday insecurities about body shape or performance [4, 23]. Although boys and girls may internalize different body ideals, both may feel socially judged when targeted by teasing [6, 13]. This shared vulnerability may explain why teasing relates to dissatisfaction in both sexes [1, 14]. Such a perspective highlights teasing as a meaningful psychosocial correlate of BI development.

Taken together, these findings highlight the importance of considering teasing as a relevant component of adolescents' everyday social environments, particularly within school contexts [9]. Preventive efforts should move beyond a narrow focus on weight‐related stigma. Because associations were observed across all teasing domains, addressing broader patterns of peer interaction and social evaluation is essential [24]. School‐based initiatives that promote respectful peer relationships and reduce normative acceptance of appearance‐based judgment may be especially relevant. Importantly, the present results underscore that subtle and non‐verbal forms of teasing, often perceived as less severe, may nonetheless be associated with BI dissatisfaction during adolescence.

### 4.1 Implications for School Health Policy, Practice, and Equity

The present findings indicate that addressing weight‐based teasing in schools requires a shift from weight‐centered approaches toward policies grounded in safety, dignity, and inclusion. First, school policies should explicitly recognize weight and appearance as protected categories within anti‐bullying frameworks, as the enumeration of weight strengthens schools' ability to address peer abuse and reduce weight bias among educators [29]. Furthermore, institutional practices that inadvertently reinforce stigma should be reconsidered. Dress codes, for example, should be revised or eliminated because they often function as “covert tools of weight stigma,” disproportionately affecting girls and students in larger bodies [9]. Schools should also discontinue BMI surveillance practices, such as BMI report cards. Evidence shows that these strategies increase body dissatisfaction and have no demonstrated effectiveness in improving health outcomes [30]. Collectively, these policy changes can improve the school climate, which is critical because the physical and socio‐emotional environment of schools shapes health behaviors and psychological well‐being [31].

These policy changes should be accompanied by adjustments in daily educational and health practices. Physical education classes are frequently identified as high‐risk environments for teasing [30]. For this reason, educators should diversify activities, including options such as dance or yoga. They should also eliminate practices that expose or single out students' bodies, which may act as triggers for stigma [9]. Health education curricula should adopt a weight‐neutral approach, emphasizing sleep, enjoyable eating, and movement for well‐being rather than weight control or calorie counting [30]. Educators and school health professionals should also be trained to recognize subtle forms of teasing, including those occurring within friendship groups and “fat talk.” These interactions often fall outside traditional bullying definitions but can be equally harmful. Indeed, teasing should be recognized as having psychological effects comparable to physical bullying [1]. Promoting equity further requires attention to intersectional vulnerabilities and the inclusion of youth voices in decision‐making. Students in larger bodies, particularly those who also experience racial or gender marginalization, may face disproportionate disciplinary scrutiny [9]. Therefore, schools should adopt equitable screening for disordered eating and involve students with lived experiences of stigma in policy development [29, 30].

### 4.2 Limitations

Several limitations should be acknowledged. Because the study is cross‐sectional, temporal ordering cannot be established, and teasing and BI dissatisfaction may reinforce one another. All teasing experiences and BI perceptions were assessed through self‐report, which may be subject to recall bias and social desirability, particularly for socially sensitive experiences. In addition, adolescents may overestimate physical activity levels, potentially introducing measurement error. Additionally, dichotomization of teasing items may have reduced variability and obscured potential dose–response relationships. Regarding the study sample, the fact that research was conducted exclusively among public high school students in Boa Vista, Roraima, may limit the generalizability of our findings to other regions. This geographical scope is further restricted by the exclusion of pregnant adolescents and those unable to complete the questionnaires independently.

Despite these limitations, the study has important strengths. It used validated instruments to assess teasing and BI, objectively measured nutritional status, and applied analytical models adjusted for key sociodemographic, behavioral, and nutritional covariates. Beyond these methodological strengths, the study contributes to the literature by examining distinct forms of teasing rather than treating teasing as a single construct, including the understudied domain of mockery related to poor motor coordination. It also distinguishes dissatisfaction due to overweight from dissatisfaction due to thinness, allowing a more nuanced understanding of adolescents' BI concerns. Finally, by examining adolescents from Northern Brazil, the study provides evidence from a Latin American context that remains underrepresented in research on teasing and BI.

## 5 Conclusions

This study showed that multiple forms of teasing, including appearance‐related, weight‐related, and performance‐related teasing, are associated with BI dissatisfaction due to both overweight and thinness among adolescents. These associations were observed for boys and girls, indicating that teasing represents a relevant correlate of BI dissatisfaction across sexes. By distinguishing forms of teasing while demonstrating a convergent pattern of associations, the findings suggest that exposure to body‐directed peer evaluation may represent a transversal psychosocial correlate of BI dissatisfaction in adolescents, extending prior research beyond content‐specific interpretations. Addressing teasing in adolescent social environments may be relevant for interventions aiming to promote healthier body‐related perceptions. Future longitudinal studies may help clarify temporal relationships and underlying mechanisms linking teasing experiences to BI development.

## Ethics Statement

The study followed the Declaration of Helsinki and was approved by the Research Ethics Committee of State University of Roraima (protocol 7,264,601, December 2024). Written informed consent was obtained from parents or legal guardians, and adolescents provided written assent.

## Consent

Written informed consent was obtained from all participants and their legal guardians prior to data collection.

## Conflicts of Interest

The authors declare no conflicts of interest.

## Data Availability

The data for the present study are not readily available. As per the participant consent form, data are only available to the research team. Any requests for the raw data should be addressed to the corresponding author.
